# Mitochondrial Deficiencies in the Predisposition to Paraganglioma

**DOI:** 10.3390/metabo7020017

**Published:** 2017-05-04

**Authors:** Charlotte Lussey-Lepoutre, Alexandre Buffet, Anne-Paule Gimenez-Roqueplo, Judith Favier

**Affiliations:** 1 INSERM UMR970, Paris-Cardiovascular Research Center at HEGP, F-75015 Paris, France; charlotte.lussey@inserm.fr (C.L.-L.); alexander.buffet@inserm.fr (A.B.); anne-paule.gimenez-roqueplo@aphp.fr (A.-P.G.-R.); 2 Equipe Labellisée Ligue contre le Cancer, F-75015 Paris, France; 3 Faculté de Médecine, Université Pierre et Marie Curie, F-75006 Paris, France; 4 Faculté de Médecine, Sorbonne Paris Cité, Paris Descartes, F-75006 Paris, France; 5 APHP, Hôpital Européen Georges Pompidou, Service de Génétique, F-75015 Paris, France

**Keywords:** SDH, paraganglioma, pheochromocytoma

## Abstract

Paragangliomas and pheochromocytomas are rare neuroendocrine tumours with a very strong genetic component. It is estimated that around 40% of all cases are caused by a germline mutation in one of the 13 predisposing genes identified so far. Half of these inherited cases are intriguingly caused by mutations in genes encoding tricarboxylic acid enzymes, namely *SDHA*, *SDHB*, *SDHC*, *SDHD*, and *SDHAF2* genes, encoding succinate dehydrogenase and its assembly protein, *FH* encoding fumarate hydratase, and *MDH2* encoding malate dehydrogenase. These mutations may also predispose to other type of cancers, such as renal cancer, leiomyomas, or gastro-intestinal stromal tumours. SDH, which is also the complex II of the oxidative respiratory chain, was the first mitochondrial enzyme to be identified having tumour suppressor functions, demonstrating that 80 years after his initial proposal, Otto Warburg may have actually been right when he hypothesized that low mitochondrial respiration was the origin of cancer. This review reports the current view on how such metabolic deficiencies may lead to cancer predisposition and shows that the recent data may lead to the development of innovative therapeutic strategies and establish precision medicine approaches for the management of patients affected by these rare diseases.

## 1. Introduction

Paragangliomas (PGLs) are rare tumours that may occur in non-chromaffin cells of the parasympathetic ganglia, typically in the head and neck region (glomus tympanicus, glomus jugulare, carotid body). These tumours can also arise in chromaffin cells of the sympathetic nervous system in the chest, abdomen, or pelvis (organ of Zuckerkandl, urinary bladder). Pheochromocytomas (PCCs) are particular PGLs that develop in the adrenal medulla. While head and neck PGLs are usually non-functional, PCCs and functional PGLs secrete catecholamines (epinephrine, norepinephrine, dopamine) in the circulation and can induce severe lethal cardiovascular and cerebrovascular complications. The prevalence of pheochromocytomas/paragangliomas (PPGLs) in patients with hypertension consulting at general outpatient clinics is estimated at 0.2% to 0.6%, but this number is probably underestimated [1]. Most PPGLs are benign, with a 10-year overall survival rate of ~96%. However, 10% of PCC patients and up to 40% of PGL patients develop a metastatic disease with a five-year survival rate below 50%. Currently, there are no histopathological criteria to predict malignancy [2], and current treatments for the metastatic forms of the disease are generally ineffective.

Approximately 40% of patients with PPGLs carry a germline mutation in one of the 13 PPGL predisposing genes identified so far, including the *RET* proto-oncogene and the *NF1*, *VHL*, and *SDHx* genes. Other rare cases involve germline mutations in the *FH*, *TMEM127*, *MAX*, and *MDH2* genes [3,4,5,6]. In addition, somatic mutations have been reported in 30% of cases, involving the *VHL*, *RET*, *NF1*, *HIF2A*, *ATRX* and *HRAS* genes [4]. Predisposing mutations can occur in apparently sporadic tumours (12–15%) or in the context of a hereditary cancer syndrome mostly represented by the following three genetic syndromes: multiple endocrine neoplasia type 2, Von Hippel–Lindau disease, and neurofibromatosis type I [7,8]. Activating mutations in the *RET* proto-oncogene lead to multiple endocrine neoplasia type 2 (MEN2), characterized by the development of medullary thyroid carcinoma, often associated with PCCs and hyperparathyroidism. The *NF1* gene, one of the largest genes in humans (60 exons) [9], encodes neurofibromin, which is a tumour suppressor that downregulates the RAS–RAF–MAPK signalling cascade. Mutations in this gene cause neurofibromatosis type 1, also known as Von Recklinghausen disease, a frequent autosomal disorder (prevalence of 1 in 3000 to 1 in 4000 people in the general population and a high penetrance) characterized by pigmentary abnormalities and neoplastic growth of neural crest-derived cells, such as multiple dermal neurofibromas and very rarely PCCs (0.1–5.7% of patients with NF1) [10]. The Von Hippel Lindau disease is a hereditary neoplastic syndrome caused by mutations in the *VHL* tumour suppressor gene, which are responsible for a predisposition to renal cell carcinoma (RCC), retinal or central nervous system hemangioblastomas, pancreatic cysts, and PCCs [11]. VHL type 1 families have a greatly reduced risk of PCC, but can develop all the other tumour types generally associated with the disease. VHL type 2 families develop PCCs, but have either a low-risk (type 2A) or high-risk (type 2B) for RCC. VHL type 2C families have PPGLs only, without the other hallmarks of VHL disease. More recently, integrative genomic approaches identified germline mutations in two new tumour suppressor genes: *TMEM127* [12] and *MAX* [13] genes which predispose to familial, bilateral, or apparently sporadic PCCs.

In 2000, the description of the first mutations in the *SDHD* gene [14] in patients with PPGL was a major breakthrough, not only in the understanding of PPGL tumorigenesis but in cancer in general [14,15]. Indeed, *SDHx* genes were the first genes encoding a mitochondrial protein implicated in the development of cancer, supporting the hypothesis of a direct link between mitochondrial dysfunction and cancer, as proposed by Otto Warburg at the beginning of the 20th century [16] and underlying the important role of metabolism as a “new” hallmark of cancer [17].

## 2. Genetically-Determined Mitochondrial Deficiencies

*SDHx* genes (*SDHA*, *SDHB*, *SDHC*, *SDHD*, and *SDHAF2*) represent almost half of the germline mutated genes in PPGL [6] (Table 1). *SDHA-D* are nuclear genes that encode the four subunits of succinate dehydrogenase (SDH), a mitochondrial enzyme located in the inner mitochondrial membrane. It catalyses the oxidation of succinate into fumarate within the tricarboxylic acid (TCA) cycle and transfers electrons to the ubiquinone pool in the respiratory chain. Succinate dehydrogenase contains two anchorage proteins (SDHC and SDHD) and two catalytic proteins (SDHA and SDHB). SDHAF2 is responsible for the flavination of the SDHA subunit, which is essential for the assembly of the complex.

*SDHx* genes behave as tumour suppressor genes in keeping with Knudson’s “two-hit” model. Hence, patients carry a germline heterozygous loss-of-function mutation and subsequent loss of heterozygosity (LOH) at somatic level, leads to the complete inactivation of the gene [18,19]. These two events are responsible for the loss of function of succinate dehydrogenase activity leading to massive succinate accumulation in the cytoplasm [20].

Previous studies have reported that germline mutations in one of the *SDHx* genes lead to PPGL predisposition in patients [7,21,22], which is transmitted in an autosomal dominant fashion for *SDHA*, *SDHB*, and *SDHC* genes mutations and in an autosomal dominant fashion with maternal imprinting for *SDHD* and *SDHAF2* genes. In *SDHx*-related PPGL, the great majority of patient have a mutation in *SDHB* and *SDHD* genes (more than 60%), while mutation in *SDHC* and *SDHA* are rare [22,23]. Patients carrying an *SDHx* germline mutation develop PPGLs at a mean age of 36 years old, while sporadic PPGLs usually occur around 50 years old [7,8]. *SDHx* germline mutations have an incomplete penetrance, and genotype-phenotype correlations have been described. *SDHD* germline mutations have a penetrance of 86% at the age of 50 and are frequently associated with the development of multiple head and neck PGL and with family history of PPGLs in the paternal branch. In contrast, *SDHB* germline mutations have an estimated penetrance of 50% at the age of 50 [24,25], frequently predispose to abdominal PGLs, and to malignant forms of the disease in 50% of cases. On the contrary, malignant PPGLs are only found in 5% of *SDHD* or *SDHC* mutation carriers [8,25].

Germline *SDHB* mutations are found in 36% of all malignant PPGLs, and *SDHB*-related malignant PPGLs have a worst prognosis than all other types of malignant PPGL. The median overall survival of patients with an *SDHB*-related malignant PPGL is of 42 months after the diagnosis of the first metastasis, while it is of 244 months for non-*SDHB* malignant PPGL [26]. The reason for this phenotypic characteristic of *SDHB* mutants is still unclear.

Germline mutation in *SDHC* gene are rare and may be associated with any type of PPGL. Finally, *SDHA* and *SDHAF2* mutations have been described in only few patients and predispose to abdominal PPGL and head and neck PGL, respectively [27,28].

Because of the genetic complexity of PPGL and of the large number of variants of unknown significance (VUS) identified in patients, immunochemical analyses of SDHA [29], SDHB [30], and SDHD [31] are now used in pathology department worldwide to validate the genetic analyses.

Germline mutations in *SDHx* genes have also been implicated in other tumours such as renal cell carcinoma (RCC) and gastro-intestinal stromal tumour (GIST) (Table 1). In 2004, Vanharata et al. described the first cases of RCC secondary to an *SDHB* germline mutation, which co-segregated with PPGL in a family [32]. Since, *SDHx* genes mutations have been implicated in 0.05 to 0.2% of renal cancers, and *SDHB* mutations are the most frequent [33]. It had recently been recognized as a subtype of renal cancer by the World Health Organization (WHO, 2016). In this subtype of RCC, various histological types have been described (chromophobe RCC, clear cell RCC, papillary RCC, and sarcomatoïd RCC). Multiple forms are described in 30% of cases [34]. In patients carrying the *SDHB* gene mutation, the lifetime risk of RCC has been estimated as 14% [24].

The great majority of GISTs are secondary to somatic mutation in *KIT* or *PDGFRA* genes. However, 85% of paediatric forms and 15% of adult forms have a so-called Wild-Type (WT) GIST, i.e., without *KIT* or *PDFGRA* mutations [35]. In these WT GISTs, more than 85% are secondary to an *SDHx* gene mutation [36]. The great majority are germline mutation (82%) and *SDHA* mutations represent more than a half of the identified mutations. *SDHx* related GISTs are more frequent in women, are always located in the gastric wall, and are multiple in 40% of cases. Malignant evolution is observed in 45% of patients, but with an indolent evolution [36]. Recently, epimutations of the *SDHC* gene promoter have been proposed as a new mechanism of SDH loss of function in WT GIST with a negative SDHB immunochemistry and without any mutations in germline or somatic DNA [37,38]. These epimutations are germline or somatic mosaic, and represent almost the quarter of *SDHx* related GIST [36]. They are more frequent in female patients and are multiple in 70% of cases. They are characterized by an *SDHC* promoter-specific CpG island hypermethylation associated with subsequent gene silencing. Recently, a patient with multiple PPGL has been described with an epimutation of the *SDHC* promotor [39].

Additional to *SDHx* genes, other genes encoding enzymes of the TCA cycle have been implicated in tumorigenesis. Fumarate hydratase, encoded by the *FH* gene, is a TCA cycle enzyme that catalyses the step immediately following SDH in the cycle, converting fumarate into malate. Germline mutations in this tumour suppressor gene were first described in the predisposition to HRLCC syndrome (hereditary leiomyomatosis and renal cell cancer) also known as Reed Syndrome [40]. More than 70% of *FH*-mutation carriers develop cutaneous leiomyoma, and women are prone to uterine leiomyoma in more than 82% of cases. In 18% of cases, FH patients develop very aggressive papillary type II renal cancer associated with poor prognosis [41,42]. More recently, it was demonstrated that *FH* gene germline mutations can also predispose to PPGL [43] and lead to malignant or multiple forms of the disease [44,45].

Finally, Cascon et al. have recently described the first *MDH2* gene germline mutation in a patient with a multiple and malignant PGL. This gene encodes malate dehydrogenase, which converts the malate into oxaloacetate in the TCA cycle [3]. Until now, this is the only case of *MDH2* mutation ever described in the predisposition to cancer.

## 3. Tumorigenesis

In 2000, the demonstration of *SDHD* being a tumour suppressor gene showed that Warburg view was actually true, at least in these very specific cases of inherited cancer predisposition, and that a defect in a central metabolic function could be the origin of cancer. Intriguingly, genetic disorders associated with this type of dysfunction had before that, been associated with neurodegenerative diseases rather than with a proliferative phenotype. Indeed, *SDHA* and *FH* germline homozygous mutations had been shown to respectively cause Leigh syndrome, a progressive brain disorder that appears in infancy or early childhood and multiple severe neurologic abnormalities in the so-called “Fumarate hydratase deficiency”. How mutations in the same genes lead to a proliferative disease was a real surprise. One explanation is that patients affected with Leigh syndrome or fumarate hydratase deficiency carry homozygous germline mutations that maintain some residual enzymatic activity, allowing these mutations to be viable and mediating the neurologic phenotype. In contrast, mutations predisposing to cancer susceptibility are heterozygous, require a second somatic genetic event (most generally the loss of the chromosomal region harbouring the wild-type allele), and then lead to the complete and selective loss of the enzymatic activity. Recently, Lorendeau et al addressed this question and suggest that tumorigenesis requires the combine loss of SDH and complex I activity, while SDH inhibition alone would solely lead to the neurodegenerative phenotype [46].

**Figure 1:**
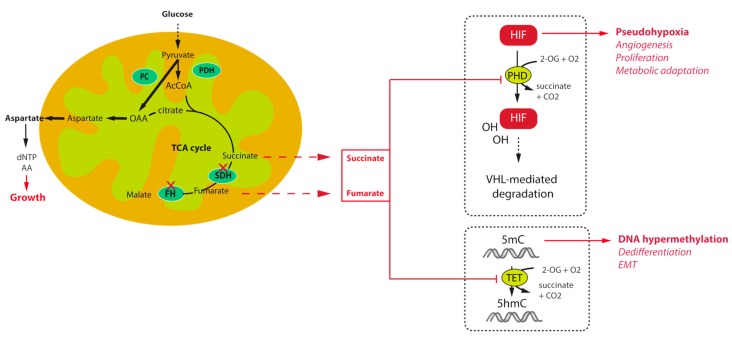
Mechanisms of tumorigenesis and survival associated with tricarboxylic acid (TCA) cycle mutations in paraganglioma.

The first key allowing understanding the tumorigenesis pathways associated with TCA cycle mutations was the description, in the first French family of *SDHD*-related PGL, of increased angiogenesis and overexpression of the hypoxia inducible factor 2α (HIF2α) and one of its target, the vascular endothelial growth factor (VEGF) [18]. Transcriptomic studies further confirmed this initial observation by demonstrating that unsupervised classification of PPGL tumours allowed separating them into two major clusters of expression: cluster 1, characterized by a hypoxic signature, which comprised all TCA cycle mutations (cluster 1A) on one side and *VHL* and *HIF2A* mutated tumours (cluster 1B) on the other side, and cluster 2, regrouping *RET*, *NF1*, *MAX*, and *TMEM127* related tumours, as well as most of the sporadic cases [47,48]. The *VHL* gene encodes an E3 ubiquitin ligase that is implicated in the ubiquitination of HIF1α and HIF2α transcription factors leading to their proteosomal degradation (Figure 1). The recognition of HIFs by pVHL requires their hydroxylation on two highly conserved proline residues by the so called HIF-prolyl hydroxylases (PHDs or EGLN) that belong to the family of 2-oxoglutarate (2-OG) dependent dioxygenases. The reaction of hydroxylation uses O_2_ as a co-substrate, explaining the stabilization of HIFs in hypoxic conditions. In VHL-mutants, loss of pVHL function leads to the abnormal stabilization of HIFs, even in the presence of oxygen, leading to a pseudohypoxic drive. In SDH and FH-deficient tumours, it was subsequently shown that the massive accumulation of their respective substrates, namely succinate and fumarate is responsible for the inhibition of PHD activity, also leading to a pseudohypoxic response. Indeed, succinate and fumarate, which are very similar to 2-OG, act as competitive inhibitors of 2-OG dependent dioxygenases. In addition, another hydroxylase is implicated in the modulation of HIF activity in normal conditions. This so-called Factor Inhibiting HIF (FIH) is an asparagyl hydroxylase that hydroxylates an asparagine residue located on the carboxy-terminal transactivation domain of HIFs, preventing the recruitment of the coactivator proteins p300 and CBP, thereby attenuating HIF-dependent transcription [49]. The inhibition of FIH activity by succinate and fumarate may as well increase the HIF-mediated transcriptional response thereby modulating the expression of a wide variety of target genes, the products of which being implicated in the regulation of angiogenesis, tumour growth, energy metabolism, survival, and migration.

Other important members of the 2-OG dependent dioxygenases family are the DNA and histone demethylases, namely the ten eleven translocation (TET) enzymes and histone lysine demethylase (KDM) family of JmjC-domain containing proteins. Oxidative demethylation of DNA is mediated by TET enzymes, which hydroxylate 5-methylcytosine (5mC) into 5-hydroxy-methylcytosine (5hmC) [50,51]. It was shown that inhibition of TET enzymes by succinate leads to a high abundance of DNA methylation and a low abundance of DNA hydroxymethylation in *SDH*-related PPGL [43] and GIST [52] (Figure 1). This has massive consequences on gene expression as hypermethylation of CpG islands within the promoter regions of genes is frequently associated with the repression of transcription. Hence, *SDH* mutant cells, by reprogramming the transcriptome at the whole genome scale, foster tumorigenesis by repressing the expression of genes that promote cancer stem cell identity [53], and Epithelial-to-Mesenchymal Transition (EMT) [54]. An EMT like phenotype has indeed been described in both *SDHB*-mutated metastatic human PPGL [55] and cells [56], as well as in FH-deficient cells [57]. This phenotype is regulated by epigenetic modifications, that comprise the hypermethylator phenotype, but also the dysregulation of key target microRNAs [4,57]. Because of their central role in the acquisition of tumour hallmarks by SDH- and FH-deficient cells, succinate and fumarate are now referred to as “oncometabolites”, as 2-hydroxyglutarate, the organic acid generated by mutant isocitrate dehydrogenase mutants in gliomas and acute myeloid leukaemia [58].

## 4. Innovative Strategies and Future Directions

As discussed earlier, *SDHx* mutation carriers are at risk of developing multiple PPGLs, and *SDHB*-mutation carriers are predisposed to metastatic forms of the disease. Malignant PPGLs respond very poorly to classical chemotherapies, and the complete surgical resection of the tumour is still the only curative treatment. For non-operable or metastatic PPGLs, therapeutic choices may take into account the particular metabolic reprogramming of these tumours.

### 4.1. New Therapeutic Strategies for SDHx-Deficient Metastatic PPGL

One promising strategy is based on an antiangiogenic approach that appears appropriate regarding the highly vascularized pattern and the activation of a VEFG-dependant angiogenesis of PPGL carrying *SDHx* mutations. The first six case reports of metastatic PPGLs treated with sunitinib were published almost simultaneously by four different teams showing extended partial response in patients carrying *SDHB* mutations [59,60,61,62]. The most comprehensive study reported so far is a retrospective review of medical records of 17 patients (including eight with an *SDHB* mutation and one *VHL* patient) with metastatic PPGLs who were treated with sunitinib [63]. Among the 14 patients without early toxicity, three had a partial response and five had stable disease, while the disease progressed in the other six patients. Partial response or stable disease were observed in the patient with a *VHL* mutation and five of the six evaluable patients with an *SDHB* mutation, suggesting that patients with cluster 1 disease might be better responders to antiangiogenic treatments than patients with cluster 2 tumours. These observations need to be confirmed in larger cohorts of patients and compared with appropriate placebo conditions. To that aim, the first randomized double blind phase II international multicentre study (the FIRSTMAPPP study) is currently ongoing and will hopefully enable such validations. The FIRSTMAPPP study is a randomized double-blind phase II international multicentre study that is evaluating the efficacy of sunitinib versus placebo in patients with progressive malignant PPGL (http://clinicaltrials.gov/ct2/show/NCT01371201), and two nonrandomized phase II studies that are evaluating the response to sunitinib (https://clinicaltrials.gov/ct2/show/NCT00843037) and axitinib (https://clinicaltrials.gov/ct2/show/NCT01967576).

Directly inhibiting HIF2α, instead of its targets like VEGF, appears to be a good strategy. Recently, two independent teams published the results of PT2399, a selective HIF-2 antagonist, in a preclinical model of clear cell renal carcinoma (ccRCC) [64,65]. Further studies are needed to evaluate if these new molecules will be effective in the PPGL field.

Other pathways identified by “OMICs” approaches are promising candidates for precision medicine. Epigenetic alterations play major roles in establishing and maintaining aberrant gene expression profiles in cancer cells and in particular in the hypermethylated cluster M1 involving all PPGL with *SDHx* and *FH* mutations [43]. Targeting epigenetic alterations in cancer cells (epigenetic therapy) is a new frontier in drug discovery. Reversion of DNA methylation by epidrugs such as 5-aza-2′-deoxycytidine (Decitabine) or histone methylation by histone methyl transferase inhibitors, is clearly an attractive strategy for carriers of *SDHx* or *FH* mutations. The efficacy of these drugs has led to their approval as monotherapy in haematological malignancies [66]. Although no study is yet available in humans, some promising results are now starting to bear fruit from in vitro studies, suggesting that drugs targeting epigenetic pathways might constitute useful alternative treatments for *SDHx*-related or *FH*-related malignant PPGLs in the future [43]. Hence, a statistically significant effect of decitabine on the cell migration was previously demonstrated in a model of hypermethylated *Sdhb*-knockout mouse chromaffin cells (ImCC) [43]. In primary cultures of human PCCs and in another PCC-derived mouse cell line (MPC), the 5-aza-cytidine DNA methylation inhibitor increased the efficacy of the proapoptotic Topoisomerase1 inhibitor (camptothecin) agent when used as a complementary drug [67].

Furthermore, the hypermethylated phenotype of *SDHB*-related tumours apparently affects the promoter of the MGMT gene encoding the O6-methylguanine-DNA methyltransferase, the repression of which is a biomarker of a good response to alkylating agents such as temozolomide [68,69]. Indeed, in the first report involving a small retrospective cohort of 15 patients with progressive metastatic PPGL, temozolomide treatment was more effective in patients carrying *SDHB* mutations than in those without *SDHB* mutations [70]. Although these data are promising, the findings will need to be confirmed in a prospective clinical trial.

### 4.2. The Explorative Way of Metabolic Reprogramming

Metabolic reprogramming is emerging as a core hallmark of cancer [71]. During the last decade, many studies have shown that the majority of oncogenes and tumour suppressor genes are involved in cell metabolism and that mutations in these genes facilitate cell survival and proliferation by promoting the use of nutrients [72]. As a central metabolic actor, alterations of enzymes in the TCA cycle result in important metabolic re-wiring in order to respond to bioenergetics and anabolic requests of cancer cells. Main changes rely on an increase of glucose and glutamine consumption and an increased anaerobic glycolysis. Hence, mutations in *SDHx* genes were demonstrated to mediate a rise in contribution of glucose to ATP production and glutamine to TCA cycle intermediates in an ovarian cancer model [73,74]. Recently, two independent studies demonstrated that loss of SDH activity through *Sdhb* deletion in a mouse cellular model results in their dependence on the pyruvate carboxylase (PC) enzyme, responsible for pyruvate carboxylation to synthesize oxaloacetate (OAA) (Figure 1). Despite the TCA cycle truncation, cells can use OAA as a TCA cycle intermediate in order to produce aspartate, a key metabolic pivot in the cell (the main precursor for protein and nucleotide biosynthesis) [75,76]. Moreover, in vitro silencing of PC resulted in complete ablation of proliferation and loss of viability [75,76]. Although it is unlikely that PC targeting would be appropriate in patients, deciphering the metabolic pathways associated with TCA cycle mutations may reveal important and novel therapeutic targets for the management of these tumours. In FH deficient renal cells, similar experiments revealed that argininosuccinate was produced from arginine and fumarate by the reverse activity of the urea cycle enzyme argininosuccinate lyase (ASL), making these cells auxotophic for arginine [77]. Fumarate also promotes protein succination that alters the function of multiple proteins, including aconitase, thus promoting to the dysregulated metabolism observed in these cells [78]. Hence, although these observations should also be confirmed in PPGL cells, they may also provide potential new therapeutic interventions or biomarkers for these cancers.

### 4.3. New Biomarkers for Diagnosis, Prognosis, and Early Response to Treatment

Increase in anaerobic glycolysis in most cancer cells and particularly in cells with an interruption of the TCA cycle lead to a wide utilisation in oncology imaging of the radionuclide ^18^F-Fluorodeoxyglucose (^18^F-FDG), a glucose analogue labelled with a positron emitter (^18^F) permitting its detection by positron emission tomography (PET) imaging. In contrast to its uptake in other neuroendocrine tumours, ^18^F-FDG uptake in PPGL provides the most sensitive functional imaging with a sensitivity on a patient basis of more than 80% [79]. Furthermore, ^18^F-FDG uptake is higher in *SDH*-deficient PGL [80,81]. ^18^F-FDG PET/CT is therefore already considered a standard examination for complete staging in patients with an *SDHx* mutation and for the evaluation of early response to treatment [82,83,84]. Nevertheless, the limitation of ^18^F-FDG PET/CT is the lack of specificity.

SDH inactivation leads to a massive accumulation of succinate, acting as an oncometabolite, and its levels, assessed on surgically resected tissues, are a highly specific biomarker of *SDHx*-mutated tumours. Succinate presents a characteristic peak at 2.44 ppm in spectral analysis that can be detected noninvasively by in vivo proton magnetic resonance spectroscopy (^1^H-MRS). ^1^H-MRS is an analytical technique for the observation of tissue compounds after elimination of water protons with a saturation pulse. Until now, ^1^H-MRS has been mostly studied in the field of cerebral tumours, especially gliomas. The majority (50–86%) of gliomas diagnosed in younger adults (<45 years old) have recurrent somatic mutations in one of the genes encoding isocitrate dehydrogenase (*IDH1* and *IDH2*) leading to the overproduction of 2-hydroxyglutarate, an oncometabolite that plays a key role in malignant transformation and is interestingly detectable in vivo by ^1^H-MRS [85,86]. A pulse ^1^H-MRS sequence was previously developed to measure succinate in an allografted mouse model of *Sdhb*-deficient tumours and used in a pilot study performed in nine patients with PPGL (five with *SDHx* mutations and four sporadic cases). That pilot study demonstrated the feasibility of detecting succinate in vivo by ^1^H-MRS as a very specific biomarker of *SDHx* mutations [87]. Moreover, animal experiments demonstrated that the area under the succinate peak of the ^1^H-MRS spectra in vivo was correlated with the concentrations of succinate measured in the resected tumours by GC-MS in vitro, allowing quantifying succinate levels in vivo. Future studies are needed to show whether this correlation holds in patients. If this turned out to be the case, and given that succinate concentrations in tumours reflect the metabolic activity of SDH-deficient tumour cells, this new innovative and innocuous imaging method could produce a quantifiable surrogate marker of early response to treatment for the patients.

## Figures and Tables

**Table 1:** Genes encoding mitochondrial proteins implicated in pheochromocytomas/paragangliomas (PPGL) susceptibility.

Genes	Enzyme	Phenotype	Mutation Frequency
*SDHA* *SDHB* *SDHC* *SDHD* *SDHAF2*	Succinate dehydrogenase	Paraganglioma/pheochromocytoma Renal cell carcinoma Gastrointestinal stromal tumour Gastrointestinal stromal tumour	20% 0.05–0.2% 12% (adult forms) 70% (paediatric forms)
*FH*	Fumarate hydratase	HRLCC syndrome Paraganglioma	70–90% 1%
*MDH2*	Malate dehydrogenase	Paraganglioma	One patient
